# At-risk and problem gambling among adolescents: a convenience sample of first-year junior high school students in Finland

**DOI:** 10.1186/s13011-015-0003-8

**Published:** 2015-03-08

**Authors:** Sari Castrén, Marjut Grainger, Tuuli Lahti, Hannu Alho, Anne H Salonen

**Affiliations:** Clinicum, Internal Medicine, University of Helsinki and Helsinki University Hospital, 00014 Helsinki, Finland; Department of Tobacco, Gambling and Addiction, National Institute for Health and Welfare, P.O. Box 30, FI-00271, Helsinki, Finland; Department of Mental Health and Substance Abuse Services, National Institute for Health and Welfare, P.O. Box 30, FI-00271, Helsinki, Finland; Faculty of Social Sciences, Department of Behavioural Sciences and Philosophy University of Turku, Turku, Finland

**Keywords:** Adolescents, At-risk/problem gambling, DSM-IV-MR-J, Gender, Smoking, Social variables, Substance use

## Abstract

**Background:**

Adolescent gambling and substance use are viewed as a public health concern internationally. The early onset age of gambling is a known risk factor for developing gambling problems later in life. The aims of this study are: to evaluate the internal consistency reliability, factorial validity and classification accuracy of the Finnish version of DSM-IV-Multiple Response-Juvenile (DSM-IV-MR-J) criteria measuring at-risk/problem gambling (ARPG); to examine gender differences in gambling participation, ARPG and substance use among first-year junior high school students; and to investigate the association of gambling and gaming (video game playing) participation, substance use and social variables with ARPG.

**Methods:**

This study examined 988 adolescents (mean age 13.4 years) at 11 public schools in Finland between October-December 2013. The response rate was 91.6%. Chi-squared test and binary logistic regression analysis were used.

**Results:**

‘Illegal acts’ was the most endorsed and sensitive, but the least specific criteria identifying ARPG. During the past year, 51.6% of the respondents had gambled, 7.9% were identified as at-risk/problem gamblers (DSM-IV-MR-J score ≥ 2), 8.0% had smoked and 8.9% had been drinking for intoxication, and the first three were significantly more common among boys than girls. The odds ratio of being a male past-year at-risk/problem gambler was 2.27, 5.78 for gambling often or sometimes, 2.42 for video game playing weekly or more often and 6.23 for having peer gamblers.

**Conclusions:**

Overall, the Finnish version of the DSM-IV-MR-J had acceptable internal consistency reliability and factorial validity. None of the DSM-IV-MR-J criteria were accurate enough to screen ARPG per se. ARPG past-year prevalence was relatively high with males gambling more than females. ARPG was as common as drinking alcohol for intoxication and smoking. Peer gambling was strongly associated with ARPG. Efficient strategies to minimise the risks of gambling problems, tools for prevention and identification of ARPG among the underage are needed.

## Background

Adolescents have an increased likelihood for developing addictive behaviours [1-3] such as gambling. Internationally, the prevalence rates of adolescent gambling problems vary from 1.6% to 6.7% [4], and these rates are higher than the rates of 0.2% to 5.3% obtained from general population samples [5]. The consequences of adolescent at-risk and problem gambling (ARPG) are multitudinous, harmfully affecting adolescents’ overall social functioning and quality of life [6]. Early onset age of gambling is associated with more severe gambling behaviour [7,8] and may predict substance use disorders, depression and other psychiatric concerns in adulthood [9]. The influence of both peers and family (e.g. family socio-demographic factors, general family climate, parenting practices, family members’ attitudes and behaviours and their relationship characteristics) is also associated with adolescent problem gambling [10-13].

In typical gambling studies gambling participation is inquired into using categorical variables measuring gambling frequency or different game type gambled, while ARPG is recommended to be measured using validated instruments [14,15]. Yet internationally, gambling studies of ARPG among adolescents have included limited investigation of instrument validity and reliability [4,14,16].

A current meta-analysis proposes that 77% to 83% of adolescents have been involved in some form of gambling [4]. Recent study from Finland revealed that 44% of adolescents had gambled during the past six months [17]. The past-year prevalence rate of Finnish population (15–74 year olds) problem gambling is 2.7% (South Oaks Gambling Screen, SOGS ≥3 points) [18] and 5.0% for adolescents (South Oaks Gambling Screen Revised for Adolescents, SOGS-RA = 2-3) [19]. Since 2006, Finnish adolescent problem gambling has been investigated by only using single questions [17,20] or extracted from population study, for example the past-year prevalence rate for 15–24 year olds was 3.8% (SOGS ≥3) [18]. In Finland many gambling opportunities are widely accessible and available, for example slot machines can be gambled at all major grocery stores and adolescents gamble despite the 18-year age limit [21]. In fact, research about the adolescent prevalence rate in Finland has been limited and the extent of this phenomenon is still unclear.

Internationally males gamble more and are at-risk/problem gamblers (ARPGers) more often than females [13,22]. Overall vulnerability of adolescents developing gambling problems may be explained by adolescents being both prone to and espousing risk-taking behaviours, and not being aware of the potential undesirable effects of such behaviours (e.g. depression, increased risk of alcohol and substance abuse disorders, increased risk of suicide ideation and attempt, higher anxiety, poor general health, disrupted familial/peer relationships, increased risk of delinquency and crime and poor academic performance) [6,13,23-25]. Phases of adolescence, especially when educational and social environments change, for example beginning junior high school, may pose a particular challenge for adopting at-risk behaviours.

In addition, the elevated rates of substance use, such as alcohol use and smoking, are associated with adolescent ARPG for both genders [26-28]. Thus substance use has been viewed as a warning sign for gambling problems and vice versa [29]. Problematic internet use, computer and/or video game playing, especially among male adolescents is also associated with gambling problems [30-35]. It has actually been proposed that video game playing shares similar features with gambling, especially with games of chance, both providing intermittent rewards to the participants [36].

Family plays a crucial role in transmitting an interest in gambling from one generation to the next [12,23,29]. Moreover, adolescent gambling frequency is related to parents’ gambling frequency and the severity of their parents’ gambling problems [37]. Furthermore, those adolescents whose parents have a positive attitude towards gambling or are problem gamblers are more likely to report ARPG themselves [12,29]. Additionally, peers play an important role in moulding the risky behaviours of adolescents. Adolescent problem gamblers tend to have peers who gamble [27,38,39] and those peers often have gambling problems [13]. Furthermore, social learning and peer modelling are strongly involved in the acquisition of gambling behaviours [36,40] and adolescents may choose a more risky behaviour when peers are actually present [41].

As several vulnerabilities and factors have been identified as being associated with adolescent gambling, adolescent gambling has been internationally recognised as a public health concern [4,42]. However, little is still known about adolescent gambling in Finland. In order to start planning harm minimization and prevention programs in Finland, the involvement and prevalence of adolescent gambling and ARPG as well as other related factors should be examined.

### Aims

In our study, the DSM-IV-Multiple Response-Juvenile (DSM-IV-MR-J) instrument was used in the Finnish context for the first time. Therefore, the first aim of this study was to evaluate the internal consistency reliability, factorial validity and classification accuracy of the Finnish version of DSM-IV-Multiple Response-Juvenile (DSM-IV-MR-J) criteria. The best DSM-IV-MR-J items would correctly identify the largest proportion of ARPGers. To be precise, the most effective items are those with high sensitivity (e.g. true positives identified correctly) and high specificity (e.g. low proportion of false positives) [43]. The second aim was to examine gender differences in gambling participation, ARPG and substance use among first-year junior high school students in Finland, since both gender differences [13,22,44] and substance abuse [24,45] are known vulnerability factors for adolescent problem gambling. The third aim was chosen based on findings of previous studies where video game playing [30-35] and family members’ and peers’ gambling [10-13] have been positively associated with adolescent problem gambling. Thus, the aim was to investigate the association of gambling and gaming (video game playing) participation, substance use and social variables with ARPG.

## Methods

A convenience sample of 1079 junior high school students from Finland was invited to this cross-sectional study. Eligible schools were recruited via the Association of Finnish Principals in September 2013. Inclusion criteria of the schools were to have at least two first-year junior high school classes in the same school and the capacity to guarantee the anonymity of the participants. Eventually, 11 schools covering the largest cities in East, North and West Finland were willing to participate in the study. Two provinces required a municipal school administration’s permission, which was applied for and granted. The Ethics Committee of the University of Helsinki approved this study.

The data were collected between October and December 2013. An information letter about the upcoming study was sent to the teachers and via the teachers to the parents. The students were then informed using a letter attached to the questionnaire. Information for parents and students included the purpose of the study and its nature being anonymous and voluntary. The questionnaire was handed out and completed in the classroom, supervised by the teachers. The students were instructed to return the questionnaire in a sealed envelope to guarantee their anonymity. In addition, the students were offered the possibility of contact with a psychologist in case they had questions or concerns regarding the study.

Ultimately, the response rate was 91.6%. Of the 988 respondents, half (50.0%) were boys, 46.8% were girls and 3.2% did not report their gender. The respondents’ age ranged from 12 to 15 years (Mean age 13.41 years; SD = 0.37). In this study the respondents are referred to as first-year junior high school students because they had just started in a new school setting. In Finland students start school at the age of seven and the targeted 7th grade students are typically aged around 13 years (corresponding US 8th grade; UK 9th grade).

### Questionnaire

The data were collected using a structured questionnaire including questions measuring gambling behaviour, substance use, video game playing and social variables (family members’ and peers’ gambling). In addition, age and gender were inquired.

#### Gambling behaviour

The gambling participation was inquired with the question: “Have you gambled?” during the past year with the following answering options: 1 = never, 2 = once or twice, 3 = sometimes and 4 = often. The names for these categories were adopted from the DSM-IV-MR-J [46]. Combining the last two categories (3 and 4) follows the example of the scoring of the DSM-IV-MR-J item numbers 2–6. Furthermore, the number of responses in two cells limited the use of the original four subcategories. Therefore, for the multivariate model the first two and the last two categories were combined.

At-risk/problem gambling during the past year was measured by the DSM-IV-Multiple Response-Juvenile (DSM-IV-MR-J) instrument including nine items [46]. The response categories included: 1 = never, 2 = once or twice, 3 = sometimes and 4 = often. Scoring of criteria 1: often = 1 point, criteria 2–6: sometimes and often = 1 point, and criteria 7–9: once or twice, sometimes and often = 1 point. Total score (range 0–9) was calculated by summing up the scores of all items. The total DSM-IV-MR-J score of two or more points was used to identify ARPGers [44].

The DSM-IV-MR-J instrument has been widely used and shown to be reliable and valid among adolescents. In the original version of the DSM-IV-MR-J, Cronbach’s alpha value was 0.75 [46] and translated versions have also shown acceptable validities and reliabilities (e.g. [47–48]). The Finnish version of the DSM-IV-MR-J was translated by a group of gambling researchers and checked by a multi-professional panel (N = 5). The back-translated Finnish version of adult DSM-IV criteria and previous literature were used as a reference [47-49].

#### Substance use

Substance use including alcohol use and smoking is associated with ARPG [45,50,51]. Alcohol use was assessed using a question: “Have you ever drunk for intoxication?” while smoking was inquired using a question: “Do you smoke?” The response options for both questions included: 1 = yes and 2 = no. The reason for using those questions in particular was to compare our results with the Finnish School Health Promotion Study [52] where the same questions were used.

#### Gaming

Gaming (video game playing) is associated with ARPG [35,36]. Video game playing was inquired with two questions: “Do you play video games at home?” and “Do you play video games elsewhere (e.g. at friends’ houses)?” with the time frame being during the past year. The response categories included: 1 = never, 2 = less than once a week and 3 = weekly or more often. First, the answers to the last two categories were combined to make a distinction between infrequent and frequent gaming. Second, the answers to both questions were combined to indicate whether the respondent had played at home and/or elsewhere.

#### Social variables

Both family [12,23,36] and peers [13,27,38,39] play a crucial role in transmitting an interest in gambling to adolescents. Social variables were inquired with three questions: *“*Do your parents gamble?”, “Do your siblings gamble?” and “Do your friends gamble?” The response categories for each question were: 1 = yes and 2 = no. The answers to the first two questions were combined to indicate whether the parents and/or siblings of the respondent gambled, and the new variable was named family gambling.

### Statistical analysis

The data were analysed using SPSS 21.0 software (SPSS, Inc., Chicago, IL, USA). Descriptive statistics included frequencies, percentages, means and standard deviations (SD). A factor analysis was performed to test the structure of the factors of the Finnish version of the DSM-IV-MR-J among first-year junior high school students. Maximum likelihood was used as an extraction method and the factors were rotated with Varimax with Kaiser normalization. Classification accuracy included analysis of sensitivity and specificity of the DSM-IV-MR-J criteria.

Statistical significance (p) was determined by the chi-squared test and binary logistic regression analysis. The exact p-values are presented in the results to detect statistically significant differences (p ≤ 0.05). 95% confidence intervals (CI) were estimated for all identified proportions. Results of the logistic regression analyses are presented as odds ratios (OR) and their corresponding CIs. In our study, odds ratio refers to the odds of being an ARPGer (DSM-IV-MR-J score ≥ 2) given the indicated risk factor divided by the odds of being an ARPGer given no indicated risk factor, when the other risk factors held fixed; that is, specific effects of risk factors on the odds of ARPG were studied. All independent variables were included in the model simultaneously.

## Results

### Internal consistency reliability, factorial validity and classification accuracy

**Figure 1:**
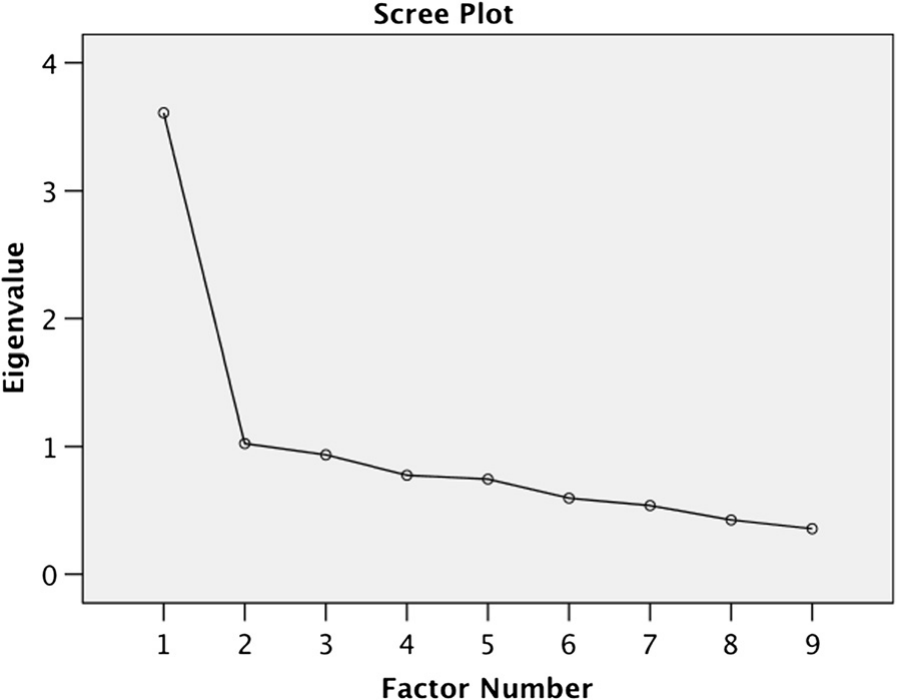
**Scree Plot of factors of the Finnish version of the DSM-IV-MR-J.**

In our study, the DSM-IV-MR-J reached the Cronbach alpha value of 0.86. Based on exploratory factor analysis, two factors with an eigenvalue over 1.0 were identified (Table 1). The first factor (eigenvalue 3.61) accounted for a larger share of the variance, since it accounted for 40.1% of variance while the other factor (eigenvalue 1.02) accounted for 11.4%. The first factor showed positive correlations with the psychological states known to be related to problem gambling: preoccupation, chasing losses, loss of control, escape and tolerance and withdrawal symptoms felt when trying to cut down on gambling. The other factor correlated with antisocial and illegal behaviours including telling lies, committing illegal or antisocial acts because of gambling and falling out with family or friends and truancy from school to gamble. Thus, the scree plot clearly supported the use of one-factor solution (Figure 1).

**Table 1:** **Factor analysis of the Finnish version of the DSM-IV-MR-J**

**Rotated factor matrix**	**n**	**Factor 1**	**Factor 2**
1. Preoccupation	983	.513	
2. Tolerance	984	.770	
3. Withdrawal	987	.511	
4. Loss of control	986	.606	
5. Escape	987	(.282)	
6. Chasing	986	.603	
7. Lies	986		.989
8. Illegal acts	984	.310	.455
9. Risked job/education/relationship	988		.357
**Rotation sums of squared loadings**			
% of variance		23.687	18.495
Cumulative % of variance		23.687	42.183
**Initial eigenvalue**			
% of variance		40.081	11.374
Cumulative % of variance		40.081	51.454

Extraction method: Maximum likelihood; Rotation method: Varimax with Kaiser Normalization; Small loadings (<0.03) were omitted; N=988.

The total number of the endorsed criteria ranged from 0 to 9 amongst both genders (Table 2). The endorsement of the DSM-IV-MR-J items varied from 0.9% (Escape) to 11.6% (Illegal acts) within all respondents. Among ARPGers the endorsement of the items varied from 10.3% (Escape) to 78.2% (Illegal acts). Sensitivity of the items varied from 0.22-0.78 while specificity varied from 0.94-0.99. Illegal acts, tolerance, loss of control and lies were not only the most commonly endorsed DSM-IV-MR-J criteria, but also the most sensitive items in identifying ARPGers among first-year junior high school students in Finland. Yet, illegal act was the least specific criteria in the DSM-IV-MR-J instrument.

**Table 2:** **Positive endorsement and classification accuracy of the DSM-IV-MR-J criteria during the past year**

**DSM-IV-MR-J**		**All respondents**	**ARPGers**	**Sensitivity**	**Specificity**
		**n=988**	**n=78**		
**Dimension**	**Criteria**	**n (%, CI)**	**n (%, CI)**		
**1. Preoccupation**	Preoccupied with gambling (e.g. thinking about gambling or planning next venture).	28 (2.8±1.0)	24 (30.8±10.3)	0.31	0.99
**2. Tolerance**	Needs to gamble with increasing amounts of money in order to achieve the desired excitement.	45 (4.6±1.3)	40 (51.3±11.1)	0.51	0.99
**3. Withdrawal**	Restlessness or irritability when attempting to cut down or stop gambling.	20 (2.0±0.9)	19 (24.4±9.5)	0.24	0.99
**4. Loss of control**	Often spent much more money on gambling than planned.	53 (5.4±1.4)	38 (48.7±11.1)	0.49	0.98
**5. Escape**	Gambles as a way of escaping from problems or relieving dysphoric mood (e.g. feelings of helplessness, guilt, anxiety, depression).	9 (0.9±0.6)	8 (10.3±6.8)	0.10	0.99
**6. Chasing**	After losing money gambling, often returns another day in order to get even (“chasing” one’s losses).	30 (3.0±1.1)	29 (37.2±10.7)	0.37	0.99
**7. Lies**	Lies to family about gambling behaviour.	41 (4.1±1.2)	38 (48.7±11.1)	0.49	0.99
**8. Illegal acts**	Committed unsocial or illegal acts, such as gambling with school dinner or fare money, stealing from home or from outside home.	115 (11.6±2.0)	61 (78.2±9.2)	0.78	0.94
**9. Risked job/education/relationship**	Has had arguments with family, friends or others, or truanted from school because of gambling.	24 (2.4±1.0)	17 (21.8±9.2)	0.22	0.99
Total score* ≥2		78 (7.9±1.7)	-	-	

*At-risk/problem gambling (ARPG) was defined using the DSM-IV-MR-J score≥2 [46]; CI, Confidence Intervals.

### Gambling participation, at-risk/problem gambling and substance use by gender

During the past year, 51.6% of the respondents had gambled once or twice, sometimes or often (Table 3). Altogether, 7.9% of the respondents were identified as past-year ARPGers (DSM-IV-MR-J ≥ 2 points). Further analysis showed that 4.9% were at-risk gamblers (DSM-IV-MR-J = 2-3 points) and 3.0% problem gamblers (DSM-IV-MR-J≥4 points). Of all respondents, 8.0% had smoked and 8.9% had been drinking for intoxication (Table 3). Past-year gambling frequency, ARPG and smoking were statistically significantly more common among boys compared with girls. There were no statistically significant gender differences in drinking for intoxication.

**Table 3:** **Gambling participation, at-risk/problem gambling and substance use among adolescents by gender**

	**All**	**Boys**	**Girls**	
	**n (%, CI)**	**n (%, CI)**	**n (%, CI))**	**Significance**
**Gambling frequency, past-year (n=956)**				Chi=77.263, df=3, p≤0.001
No gambling	463 (48.4±3.1)	185 (37.4±4.3)	278 (60.2±4.5)	
Once or twice	298 (31.2±2.9)	160 (32.4±4.1)	138 (29.9±4.2)	
Sometimes	167 (17.5±2.4)	123 (24.9±3.8)	44 (9.5±2.7)	
Often	28 (2.9±1.1)	26 (5.3±2.0)	2 (0.4±0.6)	
**Past-year at-risk/problem gambling (DSM- IV-MR-J) (n=956)**				Chi=40.892, df=1, p≤0.001
At-risk/problem gambling (score ≥2)	76 (7.9±1.7)	66 (13.4±3.0)	10 (2.2±1.3)	
No gambling or no risk/problem (score 0–1)	880 (92.1±1.7)	428 (86.6±3.0)	452 (97.8±1.3)	
**Smoking (n=946)**				Chi=4.512, df=1, p=0.041
Yes	76 (8.0±1.7)	48 (9.9±2.7)	28 (6.1±2.2)	
No	870 (92.0±1.7)	439 (90.1±2.7)	431 (93.9±2.2)	
**Drinking for intoxication (n=949)**				Chi=2.298, df=1, p=0.138
Yes	84 (8.9±1.8)	50 (10.2±2.7)	34 (7.4±2.4)	
No	865 (91.1±1.8)	440 (89.8±2.7)	425 (92.6±2.4)	

Significance (p) is determined by Chi-squared test; N=988; CI, Confidence Intervals.

### At-risk/problem gambling and the correlates

Based on bivariate analyses, gambling often or sometimes was statistically significantly associated with ARPG compared with being a non-gambler or gambling once or twice (Table 4). Of the respondents, 18.8% had played video games weekly or more often, 48.9% had played less than once a week and 34.2% had never played video games. Also video game playing weekly or more often was statistically significantly associated with ARPG compared with video game playing never or less than once a week. Both smoking and drinking for intoxication were statistically significantly associated with ARPG compared with non-smokers and respondents who had not been drinking for intoxication. Both family gambling and peer gambling were statistically significantly associated with higher proportion of ARPGers.

**Table 4:** **Association between at-risk/problem gambling and the correlates**

**Correlates**	**chi**	**df**	**p**	**ARPG associated with**	**Reference categories**
**Gambling and gaming participation**					
Gambling frequency (4 groups)	281.677	3	≤0.001	Often or sometimes	Never or once or twice
Video game playing (3 groups)	71.718	2	≤0.001	Weekly or more often	Never or less than once a week
**Substance use**					
Smoking (2 groups)	31.822	1	≤0.001	Yes	No
Drinking for intoxication (2 groups)	45.740	1	≤0.001	Yes	No
**Social variables**					
Family gambling (2 groups)	6.123	1	0.016	Yes	No
Peer gambling (2 groups)	148.075	1	≤0.001	Yes	No

At-risk/problem gambling (ARPG) was defined using the DSM-IV-MR-J criteria (score≥2); Significance (p) is determined by chi-squared test; N=988.

The multivariate model with ARPG and the correlates are presented in Table 5. The odds ratio (95% CI) of being a male past-year ARPGer was 2.27 (1.0-5.0). In practice this means that the likelihood of being a male past-year ARPGer is roughly 2 times higher for males than for females, whereas, the odds ratio for gambling often or sometimes was 5.78 (3.0-11.0) and 2.42 (1.3-4.5) for video game playing weekly or more often. Further, the odds ratio (95% CI) of being an ARPGer who had been drinking for intoxication was 2.00 (0.9-4.4), 1.74 (0.8-4.0) for smoking, 0.94 (0.5-1.7) for family gambling and 6.23 (3.8-13.8) for having peer gamblers. In the model, goodness of fit was assessed using Nagelkerke’s R^2^ which was 0.451.

**Table 5:** **Multivariate model with the correlates and at-risk/problem gambling**

	**OR**	**p**	**95% CI**
**Socio-demographic**			
Male	2.27	0.042	1.0-5.0
Female	a	a	a
**Gambling and gaming participation**			
Gambling often or sometimes	5.78	≤0.001	3.0-11.0
Never or once or twice	a	a	a
Video game playing weekly or more	2.42	0.006	1.3-4.5
Video game playing never or less than once a week	a	a	a
**Substance use**			
Drinking for intoxication	2.00	0.087	0.9-4.4
No drinking for intoxication	a	a	a
**Smoking**	1.74	0.191	0.8-4.0
No smoking	a	a	a
**Social variables**			
Family gambling	0.94	0.846	0.5-1.7
No family gambling	a	a	a
Peer gambling	6.23	≤.001	3.8-13.8
No peer gambling	a	a	a
**Nagelkerke**			.451

Binary logistic regression analysis; At-risk/problem gambling was defined using the DSM-IV-MR-J criteria (score≥2); Reference group for at-risk/problem gambling: No gambling or no risk (DSM-IV-MR-J score=0-1); N=931; CI, Confidence Interval.

## Discussion

Our study was the first in Finland using the DSM-IV-MR-J. Internal consistency reliability of the Finnish version of the DSM-IV-MR-J was acceptable and even higher than the original instrument’s alpha value [46]. Our results with DSM-IV-MR-J supported the use of the one-factor solution as with the original version [46]. However, our study is limited by the fact that we did not follow all the steps of the cross-cultural adaptation described by Beaton and colleagues [53]. Internationally, the DSM-IV-MR-J has been criticised for producing inflated prevalence rates [14,54-56]. Therefore, further investigation of classification accuracy of the Finnish version of the DSM-IV-MR-J is needed using a parallel assessment with another instrument.

In this study, illegal act, loss of control, tolerance and lies were the most sensitive DSM-IV-MR-J criteria in identifying ARPGers. Eventually, the most sensitive criteria, illegal acts, were the least specific criteria. It has been suggested that even at-risk gambling level, stealing money may be a strong early warning sign of gambling problems [44]. Conversely, illegal or unsocial acts and loss of control may also reflect adolescent age-related impetuous behaviour in general. Though the criterion of illegal acts was removed from the DSM-5 criteria for gambling disorder [49,57], since this particular measure is based on DSM-IV criteria, it was included here. Previous research indicates that pre-occupation, tolerance, escape and chasing losses are the most important indicators of adolescent problem gambling [6,23]. Studying both at-risk and probable problem gamblers as one group may explain the high endorsement of these particular criteria, since all three items have been found highly endorsed by adolescent pathological gamblers [6]. Moreover, a previous study found specific gender differences among ARPGers in endorsements [44]. Our sample size, however, did not allow us to look at the gender differences. Nevertheless, these signs are worth noticing while planning preventive and educative interventions for adolescents.

Over half of the respondents had gambled during the past year. However, gambling participation was clearly lower than in international estimates, where the gambling participation rate varies from 77% to 83% [4]. Still, the gambling participation was slightly higher compared to earlier Finnish study (e.g. [17]). The estimates of ARPG (7.9%) found in this study seems to fall within a medium range of international prevalence rates [4,44], but higher than reported earlier [18,19]. In this study gambling included lotto, slot machines, scratch cards, Toto-games, Internet gambling and other games you bet with money. The study in 2006 was the first in Finland that used a measure targeted for adolescents, the South Oaks Gambling Screen Revised for Adolescents (SOGS-RA), which may produce higher prevalence rates than DSM-IV-MR-J [58,59]. Our prevalence estimate is also higher than reported in 2011 [18], a study that used the adult version of the SOGS, which is not a recommended measure for adolescents [60]. Our results may not be directly comparable to previous Finnish studies [18,19], those being population studies with more reliable generalizability. The prevalence estimates of this study are also higher than estimates from other Nordic countries [16,58,61-63], which may be explained by Finland’s greater availability and accessibility to gambling activities, or positive attitudes towards gambling especially among Finnish males [64].

Both gambling frequency and ARPG were greater among males than females as noted in previous studies on both adolescents [65,66] and adults [5,65]. Our results also confirmed that male adolescents smoked more than females [52,67]. The gender difference regarding gambling may be influenced by sex-role socialisation [68], thus suggesting that there might be a similar aetiology to gambling participation as with other high-risk behaviours [69]. Male problem gamblers score higher in impulsivity, antisocial personality and sensation seeking [70,71] which may play an important role in males being more prone to ARPG behaviours.

Both underage gambling participation and the relatively high prevalence rate of problem gambling in this sample indicate that the enforcement of age limits regarding underage gambling has not been efficient [21]. Since opportunities for gambling are widely accessible and available in Finland, it may be that some adolescents find their way to get around the regulatory system, for example by using an adult’s account or by gambling in informal settings privately. Our results are alarming since the respondents were considerably younger than 18, which is the legal age for gambling in Finland.

Smoking and drinking are both well-known adolescent risky behaviours [72,73]. However, gambling has not been perceived as such a high-risk behaviour, for example by parents and teachers [74-77], as signs of gambling are not easily observable [78] and gambling being perceived as a socially acceptable form of entertainment [13]. Male adolescents are found to be prone to alcohol use and delinquency [79]. In our study, however, no statistically significant gender difference was found in drinking for intoxication. These findings warrant further investigation because both early onset age of gambling and male gender are clearly associated with substance-related problems.

Our results confirm that frequent video game playing along with frequent gambling were associated with ARPG [30,35]. Video game playing is a very popular hobby among adolescents, particularly males. In fact, video game playing includes similar elements to gambling such as loss of sensation of time, experiencing a “high” and relaxing or “escaping” [35]. Our findings highlight the importance of acknowledging the association between video game playing and ARPG for this particular age group. Video game players may think that skills that they have for video game playing also apply to gambling due to their similar structural characteristics [35]. This perception leads to an illusion of control [80], and puts adolescents in a vulnerable position in regard to gambling activities. Thus, appropriate knowledge (risks involved) should be provided to adolescents and their parents and, moreover, raise public awareness about the possible risks of excessive gambling and offer tools for early detection of problem behaviour.

Parental gambling is associated with adolescent gambling and gambling problems [23,37]. Gambling may be some type of family affair that is viewed to be far more normal and acceptable than alcohol drinking or cigarette smoking. Some parents are not too alarmed about their children gambling and many parents, in fact, gamble alongside their children [76]. Our study found peer gambling strongly associated with ARPG. Our finding is in line with Hardoon and Derevensky [13] and recent results from a Norwegian study [62]. Peers play an important part in the lives of first-year junior high school students and that may explain why the association of peer gambling was superior compared with family member gambling. Another explanation for the significant association of peer gambling may be that adolescents’ attitudes towards gambling are closely related to what their peers think about gambling.

It is noted that social learning and peer modelling are strongly involved in the acquisition of gambling behaviours [36], and adolescents may choose a more risky behaviour when peers are actually present [41]. Besides, a high level of peer influence may lower adolescents’ coping skills [81]. Thus, school-based prevention programs (e.g. Stacked Deck), where students are taught the history of gambling: the true odds and gambling-related cognitive erroneous thoughts, early signs of problem gambling, causes and risk factors of problem gambling and skills for good decision making and problem solving [82], are highly recommended for implementation internationally.

As the age group of young adolescents has previously received relatively little attention in gambling literature [4], the sample size of the current study was relatively large and response rate remarkably high. However, the sample consisted of students from eight out of eleven provinces, but lacked any schools from the south of Finland. The greater areas of South Finland’s school district do not allow any type of survey to be carried out with their students or teachers. However caution is needed regarding the generalizability of the results. Future studies on population-based prevalence of ARPG of the target age group are needed.

The strength of the study is that risky behaviours other than gambling were included, which allows for identifying common risk factors and thus individuals who may be predisposed to developing risky behaviour in different domains. Still, some variables (i.e. smoking and alcohol drinking) were assessed with single questions, which may be viewed as subjective measures. Thus, our results related to the prevalence of smoking and alcohol use are consistent with the Finnish School Health Promotion Study [52]. It is also worth mentioning that the use of a single question inquiring into drinking for intoxication and smoking had possibly affected why they were found significant in the bivariate analyses, and non-significant in multivariate analyses where other factors overpowered their influence. Nevertheless, future studies may address this issue by using questions that clearly specify, for example, the frequency of use (e.g. weekly, daily, occasionally), the time frame, what was smoked (cigarette, marijuana), and more specific questions concerning alcohol use by using more clear categories (mild, moderate, heavy), or using, for example, the existing Adolescent Alcohol Involvement Scale (AAIS) [83].

Finally, the potential influence of peers should also be noted. Adolescents may overestimate their peers’ gambling and these misperceptions may affect their actions [84,85]. Herein it is possible that the respondents overestimated their own gambling activity to give socially acceptable answers among peers. It is also possible that peer influence had been present during the completion of the questionnaire, since the participants filled the questionnaire in their classrooms supervised by their respective teachers. To overcome this assumption, supervision of the participants should be more controlled.

## Conclusions

Overall, the Finnish version of the DSM-IV-MR-J had acceptable internal consistency reliability and factorial validity. None of the DSM-IV-MR-J criteria were accurate enough to screen ARPG per se. ARPG past-year prevalence was relatively high with males gambling more than females. ARPG was as common as drinking alcohol for intoxication and smoking. Peer gambling was strongly associated with ARPG. Therefore, ARPG among Finnish adolescents is an area of public health concern for Finnish policy makers, which must be addressed with further studies to elucidate the extent and severity. Currently there are no prevention programs or specific treatment facilities for youth gambling in Finland. Efficient strategies to minimise the risks of gambling problems, tools for prevention and identification of ARPG among the underage are needed. Therefore, specific courses of actions should take place.

First, the laws prohibiting the underage from gambling must be rigorously enforced. Further studies should also examine how efficient these laws and plans can be. Second, more resources and funding for prevention programs at schools are needed. Third, more efforts are needed to increase the public awareness of gambling being a type of adolescent high-risk behaviour, along with substance abuse, that requires attention. Fourth, legislators and policy makers should be properly informed regarding the importance of educating the public about responsible gambling practices.

As an example, a clear public health policy, Framework for Action [42] for youth gambling from the International Centre for Youth Gambling Problems and High-Risk Behaviours, Montreal, Canada, has four goals to protect the underage from gambling-related harms: a) de-normalization of youth problem gambling (challenging myths, and misconceptions about youth gambling and promoting realistic and accurate knowledge of the impact of youth gambling), b) prevention, c) protection (protect children and adolescents from potentially harmful products) and d) harm reduction, which focuses on preventing the specific problem behaviour from developing (especially for those who are at risk) [42].

## References

[1] Forrest D, McHale IG (2012). Gambling and problem gambling among young adolescents in Great Britain. J Gambl Stud.

[2] Steinberg L (2010). A dual systems model of adolescent risk taking. Dev Psychobiol.

[3] Casey B, Jones RM (2011). Breaking and accelerating of the adolescent brain. J Res Adolesc.

[4] Blinn-Pike L, Worth S, Jonkman J (2010). Adolescent gambling: a review of the emerging field of research. J Adol Health.

[5] Hodgins D, Stea J, Grant J (2011). Gambling disorders. Lancet.

[6] Derevensky JL, Gupta R, Winters K (2003). Prevalence rates of youth gambling problems: are current rates inflated?. J Gambl Stud.

[7] Burge AN, Pietrzak RH, Molina CS, Petry NM (2004). Age of gambling initiation and severity of gambling and health problems among older adult problem gamblers. Psych Serv.

[8] Granero R, Penelo E, Stinchfield R, Fernandez-Aranda F, Savvidou LL, Fröberg F (2014). Is pathological gambling moderated by age?. J Gambl Stud.

[9] Grant JE, Potenza MN, Weinstein A, Gorelick DA (2010). Introduction to behavioral addictions. Am J Drug Alc Ab.

[10] McComb JL, Sabiston CM (2010). Family influences on adolescent gambling behavior: a review of the literature. J Gambl Stud.

[11] Smith AR, Chein J, Steinberg L (2014). Peers increase adolescent risk taking even when the probabilities of negative outcomes are known. J Dev Psych.

[12] Wickwire EM, Whelan JP, Meyrs AW, Murray DM (2007). Environmental correlates of gambling behavious in urban adolescents. J Abnorm Psych.

[13] Hardoon KK, Derevensky JL (2002). Child and adolescent gambling behaviour: current knowledge. Clin Chil Psych and Psychiatr.

[14] Stinchfield R (2010). A critical review of adolescent problem gambling assessment instruments. Intern J Adolesc Med Health.

[15] Williams RJ, West, BL, Simpson RI. Prevention of problem gambling: a comprehensive review of the evidence, and identified best practices. Report prepared for the Ontario Problem Gambling Research Centre and the Ontario Ministry of Health and Long Term Care. October 1, 2012. http://hdl.handle.net/10133/3121.

[16] Kristiansen S, Jensen SM (2011). Prevalence of gambling problems among adolescents in the Nordic countries: an overview of national gambling surveys 1997–2009. Int J Soc Welfare.

[17] Raisamo S, Halme J, Murto A, Lintonen T (2013). Gambling-related harms among adolescents: a population-based study. J Gambl Stud.

[18] Turja T, Halme J, Mervola M, Järvinen-Tassopoulos J, Ronkainen JE (2012). Suomalaisten rahapelaaminen 2011. [Finnish Gambling Study 2011].

[19] Ilkas H, Aho P. Nuorten rahapelaaminen. 12-17-vuotiaiden rahapelaaminen ja peliongelmat -puhelinhaastattelu. [Adolescent gambling. 12–17 year olds’ gambling: a telephone survey]. Helsinki: Ministry of Social Affairs and Health & Taloustutkimus Ltd: 2006. p. 1–48.

[20] Järvinen-Tassopoulos J, Metso L (2009). Pojat ovat pelimiehiä, tytöt rahapelien harrastajia. Vuoden 2007 ESPAD -koululaiskyselyn tulosten tarkastelua. [Boys are gamblers and girls gamble for leisure activity: an analysis of your 2007 ESPAD school survey]. Yhteiskuntapolitiikka.

[21] Warpenius K, Holmila M, Raitasalo K (2012). Peliin ei puututa. Alkoholin, tupakan ja rahapeliautomaattien ikärajavalvontaa testanneet ostokokeet vähittäisliikkeissä. [Enforcing age limits on purchaces on alcohol, and tobacco and the use of slot machines: test purchases in retail outlets]. Yhteiskuntapolitiikka.

[22] Jackson AC, Dowling N, Thomas SS, Bond L, Patton G (2008). Adolescent gambling behaviour and attitudes in an Australian population: intern. J Ment Health Addict.

[23] Winters KC, Stichfield RD, Botzet A, Anderson N (2002). A prospective study on youth gambling behaviors. Psych Addict Behav.

[24] Potenza MN, Fiellin DA, Heninger GR, Rounsaville BJ, Mazure CM (2002). Gambling: an addictive behavior with health and primary care implications. J Gen Intern Med.

[25] Noel X (2013). Why adolescents are at risk of misusing alcohol and gambling. Alcoh Alcoholism.

[26] Desai RA, Maciejewski PK, Pantalon MV, Potenza MN (2005). Gender differences in adolescent gambling. Annal Clinic Psych.

[27] Hurt H, Giannetta JM, Brodsky NL, Shera D, Romer D (2008). Gambling initiation in preadolescents. J Adolesc Health.

[28] Barnes GM, Welte JW, Hoffman JH, Tidwell MC (2009). Gambling, alcohol, and other substance use among youth in the United States. J Stud Alc Dr.

[29] Shead NW, Derevensky JL, Gupta R (2010). Risk factors associated with youth problem gambling. Int J Adolesc Med Health.

[30] Parker DA, Taylor RN, Eastabrook JM, Schell SL, Wood LM (2008). Problem gambling in adolescence: relationship with internet misuse, gaming abuse and emotional intelligence. Pers Indiv Differ.

[31] Delfabbro P, King D, Lambos C, Puglies S (2009). Is video-game playing a risk factor for pathological gambling in Australian adolescents?. J Gambl Stud.

[32] Brezing C, Derevensky JL, Potenza MN (2010). Non-substance-addictive behaviors in youth: pathological gambling and problematic Internet use. Child Adolesc Psychiatr Clin N Am.

[33] Griffths M, Wood RTA (2000). Risk factors in adolescence: the case of gambling, videogame playing, and the internet. J Gambl Stud.

[34] Gupta R, Derevensky JL (1996). The relationship between gambling and video-game playing behavior in children and adolescents. J Gambl Stud.

[35] Wood RTA, Gupta R, Derevensky JL, Griffths M (2004). Video game playing and gambling in adolescents: common risk factors. J Child Adol Subst Ab.

[36] Gupta R, Derevensky JL (1998). Adolescent gambling behavior: a prevalence study and examination of the correlates associated with problem gambling. J Gambl Stud.

[37] Vachoon J, Vitaro F, Wanner B, Tremblay RE (2004). Adolescent gambling: relationships with parent gambling and parenting practices. Psych Addict Behav.

[38] Delfabbro P, Thrupp L (2003). The social determinants of youth gambling in South Australian adolescents. J Adolesc.

[39] Donati MA, Chiesi F, Primi C (2013). A model to explain at-risk/problem gambling among male and female adolescents: gender similarities and differences. J Adolesc.

[40] Gupta R, Derevensky JL (1997). Familial and social influence on juvenile gambling behavior. J Gambl Stud.

[41] Blakemore SJ, Robbins TW (2012). Decision-making in the adolescent brain. Nat Neurosci.

[42] Messerlian C, Derevensky JL, Gupta R (2005). Youth gambling problems: a public health perspective. Health Promo Int.

[43] Kuhn M, Johnson K (2013). Applied predictive modeling.

[44] Ellenbogen S, Derevensky JL, Gupta G (2007). Gender differences among adolescents with gambling-related problems. J Gambl Stud.

[45] Potenza M, Wareham JD, Steinberg MA, Rugle L, Cavallo DA, Krishnan-Sarin S (2011). Correlates of at risk/problem internet gambling in adolescents. J Am Child Adolesc Psych.

[46] Fisher S (2000). Developing the DSM-IV- criteria to identify adolescent problem gambling in non-clinical populations. J Gambl Stud.

[47] Pajula M. Pelin merkit. Tietoa rahapeliongelmasta työssään peliongelmia kohtaaville [Information booklet about problem gambling]. National Institute for Health and Welfare 2009. https://www.julkari.fi/handle/10024/90823.

[48] Castrén S, Salonen AH, Alho H, Lahti T (2014). Challenges in translating DSM-5 criteria for gambling disorder into Finnish. NAD Commentary.

[49] Castrén S, Salonen AH, Alho H, Lahti T (2014). Rahapeliriippuvuuden diagnostiikka muutoksessa. [Gambling Disorder in DSM-5 classification, a review article]. Suom Laakaril.

[50] Lorains FK, Colishaw S, Thomas SH (2011). Prevalence of comorbid disorders in problem and pathological gambling: systematic review and meta-analysis of population surveys. Addiction.

[51] Castrén S, Basnet S, Salonen AH, Pankakoski M, Ronkainen J-E, Alho H (2013). Factors associated with disordered gambling in Finland. Subst. Abus. Treat Prev and Polic.

[52] NIH, National Institute for Health and Welfare, Finland: School Health Promotion Study 2013 (Kouluterveyskysely 2013). Retrieved 12.4.2014 from: http://www.thl.fi/fi_FI/web/fi/tilastot/vaestotutkimukset/kouluterveyskysely/tulokset/aiheittain/paihteet_ja_riippuvuudet.

[53] Beaton D, Bombardier C, Guillemin F, Ferraz MB (2000). Guidelines for the process of cross-cultural adaptation of self-report measures. Rev Spine.

[54] Derevensky JL, Gupta R (2000). Prevalence estimates of adolescent gambling: a comparison of the SOGS-RA, DSM-IV-J, and the GA 20 questions. J Gambl Stud.

[55] Jacques C, Ladouceur R (2003). DSM-IV-MR-J criteria: a scoring error that may be modifying the estimates of pathological gambling among youths. J Gambl Stud.

[56] Ladouceur R, Ferland F, Poulin C, Vitaro F, Wiebe J (2005). Concordance between the SOGS-RA and the DSM-IV criteria for pathological gambling among youth. Psych Addict Behav.

[57] American Psychiatric Association (2013). Diagnostic and statistical manual of mental disorders.

[58] Olason DT, Sigurdardottir KJ, Smari J (2006). Prevalence estimates of gambling participation and problem gambling among 16-18-year-old Students in Iceland: a comparison of the SOGS-RA and DSM-IV-MR-J. J Gambl Stud.

[59] Skokauskas N, Burba B, Freedman D (2009). An assessment of the psychometric properties of Lithuanian Versions of DSM-IV-MR-J and SOGS-RA. J Gambl Stud.

[60] Salonen A, Castrén S, Raisamo S, Alho H, Lahti T (2014). Rahapeliriippuvuuden tunnistamiseen kehitetyt mittarit. [A review of instruments measuring gambling disorder]. Sosiaalilääketieteellinen Aikakauslehti. J Soc Med.

[61] Molde H, Pallesen S, Barton P, Hystad S, Johnsen BH (2009). Prevalence and correlates of gambling among 16 to 19-year-old adolescents in Norway. Scand J Psychol.

[62] Hanss D, Mentzoni RA, Blaszczynski A, Molde H, Torsheim T, Pallesen S. Prevalence and correlates of problem gambling in a representative sample of Norwegian 17-Year-Olds. J Gambl Stud. 2014. E-pub: doi:10.1007/s10899-014-9455-4. 10.1007/s10899-014-9455-4 PMC4534503 24619792

[63] Abbott MW, Volberg R, Rönnberg S (2004). Comparing the New Zealand and Swedish national surveys of gambling and problem gambling. J Gambl Stud.

[64] Salonen AH, Castrén S, Raisamo S, Orford J, Alho H, Lahti T (2014). Attitudes towards gambling in Finland: a cross sectional population study. BMC Public Health.

[65] Jacobs DF, Derevensky JL, Gupta R (2004). Youth gambling in North America: long term trends, future prospects. Gambling problems in youth: developmental and applied perspectives.

[66] Chaumeton NR, Ramowski SK, Nystrom RJ (2011). Correlates of gambling among eighth-grade boys and girls. J Sch Health.

[67] Leeman RF, Hoff RA, Krishnan-Sarin S, Patock-Peckham JA, Potenza MN (2014). Impulsivity, sensation-seeking, and part-time job status in relation to substance use and gambling in adolescents. J Adolesc Health.

[68] Wolfgang AK (1988). Gambling as a function of gender and sensation seeking. J Gambl Behav.

[69] van Hamel A, Derevensky J, Takane Y, Dickson L, Gupta R (2007). Adolescent gambling and coping within a generalized high-risk behavior framework. J Gambl Stud.

[70] Ladd GT, Petry NM (2002). Gender differences among pathological gamblers seeking treatment. Exp Clin Psychopharmacol.

[71] Liu W, Lee GP, Goldweber A, Petras H, Storr CL, Ialongo NS, Martins SS (2013). Impulsivity trajectories and gambling in adolescence among urban male youth. Addiction.

[72] Huurre T, Lintonen T, Kiviruusu O, Aro H, Marttunen M (2011). Nuoruusiän runsaan alkoholinkäytön pitkäaikaisvaikutukset aikuisiän psykososiaaliseen hyvinvointiin. [Long-term effects of adolescent excessive alcohol use on psychosocial well-being in adulthood]. Yhteiskuntapolitiikka.

[73] Raitasalo K, Huhtanen P, Ahlstom S (2012). Nuorten päihteiden käyttö sekä käsitykset niihin liittyvistä riskeistä ja saatavuudesta. Suomen ESPAD-aineiston tuloksia 1955–2011. [Adolescent’s substance use and perceptions of their risk and availability: results from Finnish ESPAD-data from 1955–2011]. Yhteiskuntapolitiikkaa.

[74] Ladouceur R, Ferland F, Cote M-A, Vitaro F (2004). Teachers knowledge and training needs regarding youth gambling. Sch Psych Internat.

[75] Graham A, Phelps R, Maddison C, Fitzgerald R (2011). Supporting children’s mental health in schools: teacher views. Teach Teach Theor Prac.

[76] Campbell C, Derevensky J, Meerkamper E, Cutajar J (2011). Parent’s perceptions of adolescent gambling: a Canadian national study. J Gambl Is.

[77] Derevensky JL, St-Pierre R, Temcheff C, Gupta R (2014). Teacher awareness and attitudes regarding adolescent risky behaviours: is adolescent gambling perceived to be a problem?. J Gambl Stud.

[78] Derevensky JL (2012). Teen gambling: understanding a growing epidemic.

[79] Griffin K, Botvin G, Scheier L, Doyle M, Williams C (2003). Comon predictors of cigarette smoking, alcohol use, aggression, and delinquency among inner minority youth. Addict Behav.

[80] Langer EJ (1975). The illusion of control. J Pers Soc Psyc.

[81] Betancourt LM, Brodsky NL, Brown CA, McKenna KA, Gianetta JM, Yang W (2012). Is executive cognitive function associated with youth gambling?. J Gambl Stud.

[82] Williams RJ, Wood RT, Currie SR (2010). Stacked deck: an effective, school-based program for the prevention of problem gambling. J Prim Prev.

[83] Mayer J, Filstead WJ (1979). The adolescent alcohol involvement scale: an instrument for measuring adolescents’ use and misuse of alcohol. J Stud Alc.

[84] Berkowich AD. The social norms approach: theory, research and annotated bibliography. 2003. Retrieved 25.5.2014:http://alanberkowich.com/articles/social_norms.pdf.

[85] Raisamo S, Lintonen T (2012). Misperceptions of peer gambling norms in adolescents: analysis of a national sample in Finland. Open J Prev Med.

